# Fatal acute respiratory distress syndrome caused by blastomycosis after recent orthotopic liver transplantation in a non-endemic area

**DOI:** 10.1016/j.mmcr.2022.07.003

**Published:** 2022-08-10

**Authors:** Hiroshi Sogawa, Ryosuke Misawa, Roxana Bodin, David C. Wolf, Rajat Nog, George Kleinman, Seigo Nishida, Oleg Epelbaum

**Affiliations:** a Division of Intra-abdominal Transplant, Department of Surgery, Westchester Medical Center/New York Medical College, 100 Woods Road, Valhalla, NY, 10595, USA; b Section of Hepatology, Division of Gastroenterology, Department of Medicine, Westchester Medical Center/New York Medical College, 100 Woods Road, Valhalla, NY, 10595, USA; c Section of Transplant Infectious Disease, Division of Infectious Disease, Department of Medicine, Westchester Medical Center/New York Medical College, 100 Woods Road, Valhalla, NY, 10595, USA; d Department of Pathology, Westchester Medical Center/New York Medical College, 100 Woods Road, Valhalla, NY, 10595, USA; e Division of Pulmonary, Critical Care, and Sleep Medicine, Department of Medicine, Westchester Medical Center/New York Medical College, 100 Woods Road, Valhalla, NY, 10595, USA

**Keywords:** Blastomycosis, Pneumonia, Acute respiratory distress syndrome, Extracorporeal membrane oxygenation, Liver transplantation

## Abstract

In blastomycosis, immunosuppression such as that following solid organ transplantation appears to be a risk factor for the development of overwhelming lung infection fulfilling criteria for the acute respiratory distress syndrome. Our transplant center, located outside traditional endemic areas for *Blastomyces* spp, experienced a case of fatal acute respiratory distress syndrome secondary to blastomycosis pneumonia in a recipient of recent orthotopic liver transplantation. The patient expired despite support with veno-venous extracorporeal membrane oxygenation.

## Highlights

• Immunosuppression is associated with overwhelming pneumonia and lung injury in blastomycosis.
• Acute respiratory distress syndrome from blastomycosis poses a danger to recipients of solid organ transplantation.
• We describe a fatal case of ARDS from blastomycosis in a recipient of recent orthotopic liver transplantation in a non-endemic region.
• Pre-transplant lung nodules could represent asymptomatic blastomycosis that could become overt after immunosuppression.
• This case underscores the need to evaluate suspicious lung nodules prior to transplantation, resorting to biopsy if required.

## 1 Introduction

*Blastomyces* spp can be considered the most geographically widespread of the endemic mycoses in the United States, where its endemic area encompasses the Midwest (e.g., Wisconsin), Southeastern states (e.g., Mississippi), and also swings east along the St. Lawrence River valley between the United States and Canada. More recently, clustering of cases has been reported as far south in New York State (NYS) as the capital region around Albany, which is a mere 150 miles from Times Square in New York City (NYC) [1]. In such “newer” endemic areas, blastomycosis can present a particular problem for solid organ transplant (SOT) programs because lower clinical suspicion may delay initiation of therapy in contrast to cases encountered in “traditional” endemic areas. There is reason to believe that SOT recipients are prone to fulminant and lethal pulmonary blastomycosis that fulfills criteria for the acute respiratory distress syndrome (ARDS), which means that therapeutic delays may be more critical in this population than they are in immunocompetent hosts [2]. Herein we present the case of a liver transplantation recipient from our center in the immediate northern suburbs of NYC who succumbed to fatal ARDS caused by *Blastomyces* spp during the index hospitalization for his transplant despite initiation of veno-venous extracorporeal membrane oxygenation (V–V ECMO).

## 2 Case presentation

**Fig. 1:**
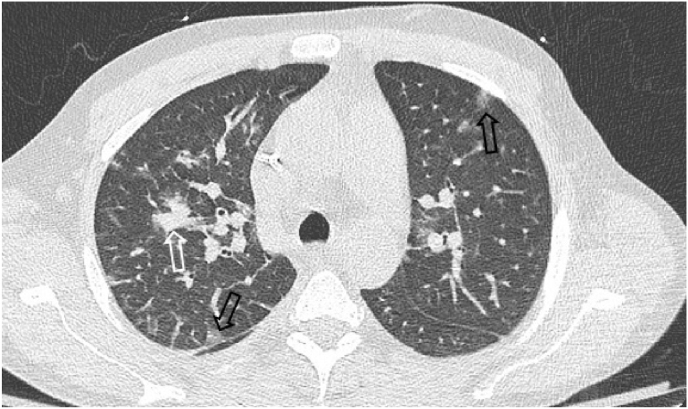
Section from chest computed tomography obtained at presentation to our institution showing bilateral upper lobe pulmonary nodules. A dominant solid nodule in the right upper lobe (white arrow) is seen among scattered sub-solid nodules (black arrows).

A 42-year-old Caucasian man with acute alcoholic hepatitis (AAH) superimposed on alcoholic cirrhosis was transferred to our institution from his local hospital for initiation of renal replacement therapy (RRT) for acute kidney injury and for liver transplant evaluation (LTE). He was a heavy drinker of alcohol but denied smoking or using illicit drugs. He reported working as a self-employed carpentry contractor, and he lived alone in Amsterdam, NY, approximately 35 miles northwest of Albany. On arrival, he was hemodynamically stable and afebrile but had a blood leukocyte count of 23K/μL (normal range 4.5–10.8K/μL). Oxygen saturation on room air was 99%, and he did not complain of dyspnea. Baseline plain chest radiograph (CXR) was not obtained. Corticosteroids were not administered for AAH. RRT was started immediately on day 0. As part of LTE, he underwent computed tomography (CT) of the chest on day +1, which was significant for lung nodules in both upper lobes, more prominent on the right (Fig. 1). He also had a consolidation in the right lower lobe (RLL) (not shown). On day +5 he underwent bronchoscopy with bronchoalveolar lavage (BAL) of the RLL; BAL was not performed in either of the upper lobes. BAL cultures from this procedure ultimately returned negative.

On day +12, the patient was listed for liver transplant with a model for end-stage liver disease-sodium (Na-MELD) score of 40. On day +13 he underwent uneventful orthotopic liver transplantation (OLT) of a cytomegalovirus (CMV)+ 30-year-old male donor organ into a CMV- recipient using the piggyback technique. The cold ischemic time was 6 h 53 min, and the warm ischemic time was 49 min. Intraoperative immunosuppression was achieved with a total of 500 mg of methylprednisolone. He received eravacycline and micafungin perioperatively. The postoperative immunosuppressive regimen consisted of tacrolimus, mycophenolate mofetil, and tapering dosing of methylprednisolone with eventual conversion to prednisone. He was also started on prophylactic trimethoprim-sulfamethoxazole (TMP-SMX) for *Pneumocystis jirovecii* pneumonia (PJP) and valganciclovir for CMV prophylaxis. RRT was continued. Bilirubin decreased from 38 mg/dl pre-transplant to about 10 mg/dl post-transplant. He required a return trip to the operating room on day +16 for surgical evacuation of a hematoma. On day +20 he was transferred from the surgical intensive care unit (SICU) with an oxygen saturation of 97% while breathing room air.

**Fig. 2:**
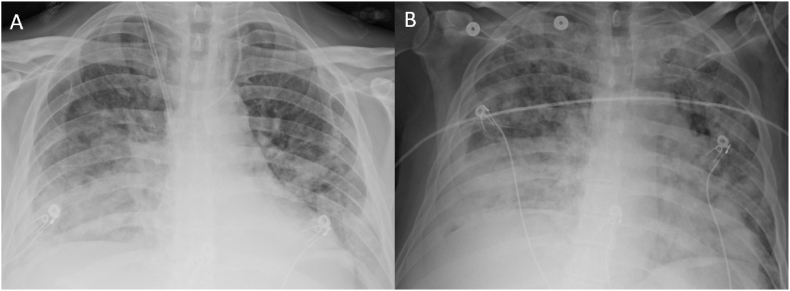
(A) Chest radiograph obtained on day +26 of admission for fever showed new bilateral airspace opacities. (B) Chest radiograph from day +32 shows interval progression of airspace disease.

On day +26, the patient was noted to have a fever and also a cough. Oxygen saturation was lower than previously at 92% on room air. CXR from that day showed new bilateral airspace opacities (Fig. 2A). He was started on broad-spectrum antibiotics and micafungin. Oxygen requirement continued to worsen along with progressively increasing serum bilirubin. Endoscopic retrograde cholangiopancreatography performed on day +27 showed no biliary stricture. A plastic bile duct stent was placed to exclude the possibility of a bile duct outflow problem. Since hyperbilirubinemia did not improve after stenting, corticosteroid dosing was increased to as much as 500 mg of methylprednisolone for three days in the setting of worsening hepatic graft function and oxygenation. On day +31 he underwent liver biopsy, which revealed bile duct inflammation with cholestasis and no rejection. Blood cultures collected on the same day grew vancomycin-resistant *Enterococcus faecium*; the patient's antibiotics were adjusted to include daptomycin. On day +32 he returned to the SICU for increasing dyspnea and persistent oxygen requirement despite achievement of net negative balance with RRT. Based on worsening airspace disease on CXR (Fig. 2B), the assumption was that the patient was still in pulmonary edema and needed more intensive RRT. Attempts to achieve this were limited by hypotension. Due to the patient's frequent receipt of blood transfusions, an additional consideration became transfusion-related acute lung injury (TRALI). Another hypothesis was daptomycin-induced pneumonitis, so antibiotic therapy was changed to tedezolid.

**Fig. 3:**
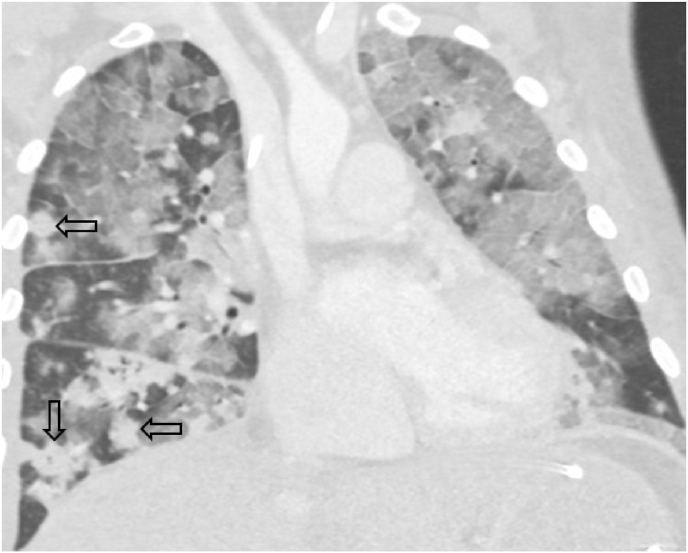
Representative coronal reconstruction image from chest computed tomography obtained following initiation of mechanical ventilation is characterized by diffuse ground-glass opacity bilaterally with intermixed nodular consolidation (arrows).

**Fig. 4:**
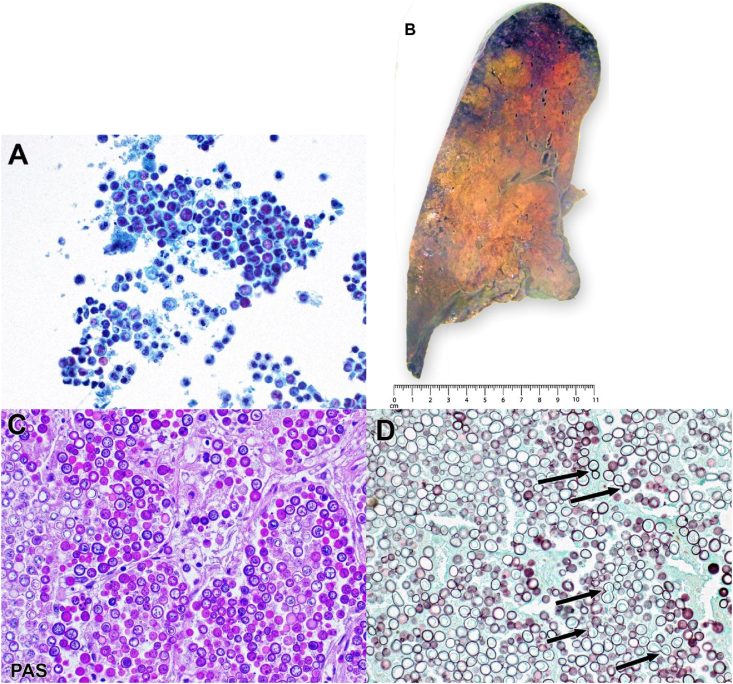
(A) Cytology specimen from bronchoalveolar lavage fluid. With the exception of a few neutrophils, it consists almost entirely of yeast forms (Papanicolaou stain; 400X). (B) Gross autopsy section of the left lung shows broad, yellow-tan areas of consolidation involving about 80% of the tissue. (C) Microscopic autopsy section of the left lung stained with Periodic acid-Schiff demonstrates near-total replacement of lung parenchyma with yeast forms exhibiting broad-based budding characteristic of *Blastomyces* spp. (200X). (D) Microscopic lung autopsy section stained with Grocott's methenamine silver highlights the broad-based budding of the pictured yeast forms (arrows, 400X). (For interpretation of the references to colour in this figure legend, the reader is referred to the Web version of this article.)

Over the subsequent 24 h he progressed to initiation of non-invasive positive pressure ventilation with an FiO_2_ of 100%. On day +33 he was endotracheally intubated, requiring persistently high FiO_2_ on invasive mechanical ventilation (≥80%) and also high-dose norepinephrine infusion. Repeat chest CT showed diffuse intermixed dense consolidation and ground glass opacities bilaterally with discernible areas of discrete nodularity (Fig. 3). TMP-SMX was increased to treatment dose for coverage of PJP. Worsening gas exchange prompted initiation of V–V ECMO on day +34 followed by bronchoscopy with BAL of the right lung. Cytological examination of the BAL specimen on day +35 revealed abundant fungal forms (Fig. 4A). Over the ensuing four days, the patient experienced continued deterioration of multiorgan failure despite maximal support and initiation of Amphotericin B, leading to family decision against further aggressive measures. On day +39 the patient expired. At autopsy, the lungs weighed >2 kg each (normal 480-570 gm) (Fig. 4B). Microscopic sections of lung demonstrated near-total replacement of lung parenchyma by yeast morphologically consistent with *Blastomyces* spp (Fig. 4C). Eventual BAL fungal culture growth further confirmed infection with *Blastomyces* spp.

## 3 Discussion

Despite growing awareness that *Blastomyces* spp is a relevant pathogen in NYS and sporadic encounters with it even in NYC and its environs [[3], [4], [5]], to our knowledge this is the first published case of blastomycosis in a SOT recipient from a transplant center located in NYS. Authors from Illinois, an endemic area, have previously reported ARDS in an OLT recipient who, like our patient, was placed on V–V ECMO and did not survive [6]. Unlike our patient's very recent transplantation (<1 month), however, OLT had occurred 15 months prior to diagnosis in this other case. A series of 30 SOT blastomycosis cases from Wisconsin, also an endemic area, included only five OLT recipients (17%), and the time from transplantation to diagnosis ranged from 5 to 66 months [2]. In another Wisconsin series that included fewer SOT patients, a similar percentage (3/19, 16%) were OLT recipients and the interval between transplantation and diagnosis was likewise much longer than in our case: 13–194 months [7].

Thus, our case is instructive not only in terms of geography but also type of SOT, development of ARDS, use of V–V ECMO, and onset of disease during the index transplant hospitalization. Of note, we concluded that V–V ECMO was of limited efficacy in this particular case because it could not overcome the high flow of the patient's own deoxygenated blood due to his hyperdynamic cardiac state in the setting of fresh liver transplantation.

*Blastomyces* spp are unique among the endemic mycoses in that extrapulmonary dissemination is common in immunocompetent hosts (40–49%) [2,7] and may paradoxically occur less often in the immunosuppressed (38%) [7]. In our patient, autopsy did reveal dissemination to the kidneys and liver, suggesting that post-OLT hyperbilirubinemia may have been an unrecognized manifestation of disseminated blastomycosis. On the other hand, overwhelming lung infection leading to ARDS and respiratory failure is relatively rare in immunocompetent hosts (10%) but quite prevalent in the immunocompromised, with SOT appearing to confer especially high risk (30–37%, OR 6.4 compared to normal immunity) [2,7]. Lung nodules, a far more common pulmonary manifestation of blastomycosis, were in fact present on the patient's pre-transplant chest CT (see Fig. 1) and likely offered an opportunity to establish the presence of *Blastomyces* spp before the patient was subjected to OLT and subsequent immunosuppression. Unfortunately, initial BAL was not directed at the location of the nodules on CT, resulting in negative cultures, and polymerase chain reaction testing was not requested. Likewise, neither antibody (sensitivity up to 64%) nor antigen (sensitivity up to 93%) testing was performed as part of serological evaluation prior to OLT [8]. This is perhaps not surprising since diagnostic delays in blastomycosis occur even in endemic areas [9] and tend to be magnified in non-endemic areas due to a lower index of suspicion [1].

The early post-transplant onset of rapidly fatal blastomycosis in this case raises the question of donor-derived infection, though this would be a rare event. More likely, our patient's disease evolved from asymptomatic pulmonary blastomycosis, a common scenario, to catastrophic pneumonia with ARDS and dissemination in the setting of therapeutic immunosuppression. It is unknown whether corticosteroid treatment, which our patient received at high doses, is of benefit in ARDS secondary to blastomycosis as has been shown for ARDS in general [10] and for ARDS due to SARS-CoV-2 in particular [11]. In a 43-patient series of blastomycosis ARDS cases not limited to SOT, corticosteroids did not impact survival but were uniformly co-administered with appropriate antifungal therapy [12]. Unopposed high-dose corticosteroid administration in ARDS of an infectious etiology could be expected to accelerate the course of the disease as may have happened in our patient. Mortality in the above series was 40%, consistent with the expected value for a cohort of predominantly severe ARDS patients [13]. Of note, all four ECMO recipients survived. The cumulative mortality of SOT recipients with blastomycosis ARDS in the two aforementioned Wisconsin series was a comparable 47% (7/15) [2,7]. Currently, guidelines do not recommend administering primary prophylaxis against blastomycosis to SOT recipients even in endemic areas, so clinical vigilance and prompt initiation of appropriate antifungal therapy are crucial for prevention of severe disease [14]. As illustrated by our case, initial deteriorations of SOT recipients in non-endemic areas tend to be addressed with echinocandins, which are not considered effective against *Blastomyces* spp.

In summary, the present case serves as a reminder that blastomycosis is a threat to SOT recipients even outside of traditional endemic areas, so it behooves clinicians in such areas to be on heightened alert for reactivation or progression of infection upon initiation of immunosuppression. SOT recipients appear to be especially vulnerable to catastrophic pulmonary blastomycosis in the form of ARDS. Transplantation candidates from regions in which *Blastomyces* spp could be reasonably encountered who are found to have pulmonary nodules on pre-transplant imaging should undergo thorough evaluation for this infection that includes CT-directed lung sampling.

## Declaration of competing interest

The authors have no conflicts of interest to disclose.
